# Lower Extremity Amputations Among Patients with Diabetes Mellitus: A Five-Year Analysis in a Clinical Hospital in Bucharest, Romania

**DOI:** 10.3390/medicina60122001

**Published:** 2024-12-04

**Authors:** Emilia Rusu, Eduard Lucian Catrina, Iulian Brezean, Ana Maria Georgescu, Alexandra Vișinescu, Daniel Andrei Vlad Georgescu, Chivu Anda Mioara, Grațiela Maria Dobra, Ioana Verde, Silviu Stanciu, Andrada Coșoreanu, Florin Rusu, Andra Nica, Doina Andrada Mihai, Gabriela Radulian

**Affiliations:** 1 Department of Diabetes, Nutrition and Metabolic Diseases, “Carol Davila” University of Medicine and Pharmacy, Malaxa Clinical Hospital, 022441 Bucharest, Romania; emilia.rusu@umfcd.ro (E.R.); alexandra.visinescu94@drd.umfcd.ro (A.V.); andrada.cosoreanu@rez.umfcd.ro (A.C.); andra.nica@drd.umfcd.ro (A.N.); 2 Department of General Suregry, “Carol Davila” University of Medicine and Pharmacy, Cantacuzino Clinical Hospital, 030167 Bucharest, Romania; eduard.catrina@umfcd.ro (E.L.C.); iulian.brezean@umfcd.ro (I.B.); 3 Department of Diabetes, Nutrition and Metabolic Diseases, Nicolae Malaxa Clinical Hospital, 022441 Bucharest, Romania; ana-maria.militaru@rez.umfcd.ro (A.M.G.); daniel-andrei-vlad.georgescu@rez.umfcd.ro (D.A.V.G.); 4 Department of Diabetes, Nutrition and Metabolic Diseases, “Prof. Dr. Nicolae Paulescu” National Institute for Diabetes, Nutrition and Metabolic Diseases, 030167 Bucuresti, Romania; anda-mioara.chivu@rez.umfcd.ro (C.A.M.); gratiela-maria.hernest@drd.umfcd.ro (G.M.D.); andrada.mihai@umfcd.ro (D.A.M.); gabriela.radulian@umfcd.ro (G.R.); 5 Department of Internal Medicine, “Carol Davila” University of Medicine and Pharmacy, Theodor Burghele Clinical Hospital, 061344 Bucharest, Romania; 6 “Carol Davila” University of Medicine and Pharmacy, 010825 Bucharest, Romania; 7 “Doctor Carol Davila” Central Military University Emergency Hospital, 010825 Bucharest, Romania; florinrusumd@yahoo.com; 8 Department of Diabetes, Nutrition and Metabolic Diseases, “Carol Davila” University of Medicine and Pharmacy, 030167 Bucharest, Romania

**Keywords:** lower extremity amputations, diabetes mellitus, diabetes-related foot disease

## Abstract

*Background and Objectives*: Lower extremity amputations (LEAs) represent a significant health problem. The aim of our study was to analyse the type and trends of diabetes-related LEAs in patients hospitalized in one surgical centre in Bucharest between 2018 and 2021. The second aim was to assess the impact of the COVID-19 pandemic on the trends of LEAs. *Materials and Methods*: We performed a retrospective analysis of all lower limb amputations performed between 01 January 2018 and 31 December 2021 in the Department of Surgery, Dr. I. Cantacuzino Clinical Hospital, Bucharest, Romania. We evaluated demographic parameters, type of LEA, the level, the laterality and trends of the amputations, the main aetiologies leading to amputation, and the length of hospitalization. *Results*: During the study period, 1711 patients underwent an LEA. The mean age was 64.53 ± 9.93 years, 71.6% (*n* = 1481) being over 60. Men outnumbered women by a ratio of 3.62:1. The most frequent interventions were ray amputations in 41.2% (*n* = 705) of patients; then, there were amputations of the toe (20.4%, *n* = 349), transtibial amputations (18.9%, *n* = 323), transfemoral amputations (10.6%, *n* = 181), and midfoot amputations (9%, *n* = 154). Wet gangrene was the most frequent aetiology (40.9%, *n* = 699). The total number of LEAs decreased constantly throughout the analysed period, such that 616 LEAs were performed in 2018 and 323 LEAs in 2021 (*p* < 0.001). There was a statistically significant increase in the rate of major LEAs in the pandemic vs. pre-pandemic period (37% vs. 24.4%, *p* < 0.001). *Conclusions*: In our study, the total number of LEAs decreased throughout the analysed period, but there was an increase in the rate of major LEAs in the pandemic vs. pre-pandemic period. Being over 65 years of age, leucocytosis, sepsis at presentation, and diabetic polyneuropathy were important risk factors for the necessity of LEA in complicated diabetes-related foot disease.

## 1. Introduction

Lower extremity amputations (LEAs) represent an important problem in the diabetic population, being associated with lower quality of life, premature mortality, and a significant economic burden for healthcare services. The mortality at 5 years after a diabetes- related LEA speaks for itself; it exceeds 70%. This excess of mortality seems to be caused by a combination of factors, such as associated conditions and complications of advanced diabetes, lack of physical activity, and deconditioning [1].

Diabetes-related foot disease (DFD) encompasses infection, ulceration, or destruction of tissues of the foot induced by neuropathy and/or peripheral artery disease in the lower extremities of a patient with diabetes mellitus. Lower extremity amputation represents the most severe form of this spectrum, which also includes callus or ulcer of the foot or lower limb, peripheral angiopathy with or without gangrene, cellulitis, osteomyelitis, mono/polyneuropathy, or neuropathic arthropathy [2,3].

Indeed, DFD is a major public health problem, as in patients with diabetes, its prevalence varies between 4.6 and 15.1%. Moreover, DFD is the leading cause for nontraumatic lower limb amputation in the developed world, and the global annual incidence of diabetes-related minor and major amputations during the 2010–2020 period was estimated to be, respectively, 139.97 and 94.82 cases/100,000 people with diabetes [4,5,6,7,8].

The most important contributing factors to LEAs in the diabetic population are diabetic foot ulcers and low extremity peripheral arterial disease, both being parts of DFD. At least half of the foot ulcers become infected and, afterward, up to 20% of significantly infected ulcers impose the need for LEA [1].

Prevention and early recognition of aggravation should play a central role in DFD management, being essential to observe early any risk factor for a possible evolution to ulceration or amputation, such as foot deformity, peripheral neuropathy with loss of protective sensation, preulcerative callus or corn, peripheral arterial disease, poor glycaemic control, visual impairment, diabetic nephropathy, cigarette smoking, or an anterior history of ulcers [9]. Increased awareness of possible evolution to amputation would favour timely guidance to a specialized multidisciplinary team in order to perform preventive and curative management of diabetic foot disease aimed at reducing the amputation rate [10]. By bridging the gap between research findings and their clinical application, this study hypothesizes that diabetes complications and metabolic disturbances contribute to the progression of diabetes-related foot disease toward amputations.

Our study aimed to analyse the type and trends of diabetes-related LEAs in patients hospitalized in one surgical centre in Bucharest between 2018 and 2021. The second aim was to assess whether there was a significant change in the number and type of amputations (major vs. minor) during the pandemic period (2020–2021) compared to the pre-pandemic period (2018–2019) and to identify the contributing factors to any observed differences, such as delayed access to healthcare, increased incidence of severe infections, or other pandemic-related barriers.

## 2. Materials and Methods

### 2.1. Study Design, Location, and Period

We performed a retrospective analysis of all lower limb amputations performed between 1 January 2018 and 31 December 2021 in the Department of Surgery, Dr. I. Cantacuzino Clinical Hospital, Bucharest, Romania’s capital and largest city with approximately 2 million inhabitants. This analysis included all adult patients with diabetes undergoing a lower extremity amputation (LEA). We evaluated demographic parameters, type of LEA, the level and laterality of amputation and trends of the amputations performed, the presence of any chronic complication of diabetes, risk factors (hypertension, obesity, dyslipidaemia), main aetiologies leading to the indication for amputation, and the length of hospitalization. Ethical approval for this study was obtained from the Ethics Committee of the Dr. I. Cantacuzino Clinical Hospital (190/15 Jan 2024).

### 2.2. Study Population

The inclusion criteria were all patients over 18 years with diabetes (type 1 diabetes mellitus T1DM, type 2 diabetes mellitus T2DM) who underwent an LEA between 2018 and 2021 in the Department of Surgery, Dr. I. Cantacuzino Clinical Hospital. Patients without diabetes who underwent amputations during the analysed period were excluded from the study. All clinical data were collected from the clinical report form each patient’s clinical report form.

LEAs were classified as major and minor. The surgical removal of only a part or multiple parts of the lower limb proximal to the ankle joint represents a major lower extremity amputation. A minor lower extremity amputation was described as any LEA distal to the ankle joint.

### 2.3. Variables

We obtained demographic parameters (e.g., age, gender) and clinical data such as the type of LEA, the level and laterality of amputation (amputation of the toe; amputation of the toe, including the metatarsal bone; mediotarsal amputation; transmetatarsal amputation; ankle disarticulation; transmaleolar amputation of the tibia and fibula; above the knee amputation; below the knee amputation; hip amputation), main aetiologies leading to the indication for amputation, the presence of hypertension, obesity, dyslipidaemia, any chronic complication of diabetes (diabetic polyneuropathy (DPN), peripheral arterial disease (PAD), chronic kidney disease (CKD) or end-stage renal disease (ESRD), diabetic retinopathy) and medical treatment, the associated antibiotic therapy and the postoperative complications, previous vascular intervention, and length of hospitalization recorded from the clinical case report.

Paraclinical laboratory tests at admission, including white blood cell (WBC) counts, C-reactive protein (CRP), erythrocyte sedimentation rate (ESR), fasting plasma glucose (FPG), cholesterol, triglycerides, HDL-cholesterol, and renal function (creatinine, estimated glomerular filtration rate (GFRe)) were analysed.

Hypertension, dyslipidemia (hypercholesterolemia, hypertriglyceridemia, or hypo-HDL cholesterolemia) were diagnosed according to the American Diabetes Association Standards of Care for 2024. The Friedewald equation was applied to estimate the LDL-cholesterol level as follows: estimated LDL-C = [total cholesterol] − [total HDL] − [estimated very low-density lipoprotein (VLDL)]. VLDL level was calculated by dividing the total triglycerides level by 5. There was no direct LDL-C testing performed. Diabetes mellitus (DM) was described using the standard criteria (blood glucose levels, HbA1c, or the use of antidiabetic treatment) [9]. Obesity was defined using a body mass index (BMI) exceeding 30 kg/m^2^. The use of tobacco was described as present or absent. Leukocytosis was defined as a number of leukocytes in peripheral blood over >11 × 10^9^/L.

### 2.4. Statistical Analysis

Qualitative variables are presented as percentages and mean ± standard deviation (SD) for continuous normally distributed data or as median (interquartile range) for continuous abnormally distributed data. We used the Kolmogorov–Smirnov with a Lilliefors significance correction and Shapiro–Wilk statistic to assess the normality of our data. Chi-square tests were used for categorical variables, independent t-tests were used for continuous normally distributed data, and Mann–Whitney U tests were used for nonnormally distributed data. A *p*-value less than 0.05 was considered significant. Data collected were analysed using Statistical Package for Social Software (SPSS) version 19. The cohort was divided according to the year of admission and the type of amputation. All variables with a *p*-value < 0.05 in the univariate analysis were included in the multivariate logistic regression models using the backward stepwise method, and an odds ratio (OR) with a 95% confidence interval was calculated.

## 3. Results

### 3.1. General Characteristics of the Population

During the study period, between January 2018 to December 2021, 1711 patients underwent an LEA. The most frequent interventions were ray/rays amputation (*n* = 705); then, in order of frequency, the following were performed: amputation of the toe (20.4%, *n* = 349), transtibial amputation 18.9% (*n* = 323), transfemoral amputation (10.6%, *n* = 181), and transmetatarsal amputation (9%, *n* = 154). Wet gangrene was the most frequent aetiology (40.9%, *n* = 699), followed by superinfected ulcers (21.8%, *n* = 373) and ischemia (11.9%, *n* = 204). The mean age was 64.53 ± 9.93 (range 26–93) years, 71.6% (*n* = 1481) being over 60 (Table 1). Men (78.4%, *n* = 1341) outnumbered women by a ratio of 3.62:1. The majority of patients were with T2DM (98.3%, *n* = 1682), and the median duration of diabetes was 13 years (CI 95% 13.55–15.02). A total of 38.8% (*n* = 664) were current smokers. August to October was the period with the highest LEAs. The most commonly isolated microorganisms were *Staphylococcus aureus* (28.1%, *n* = 71, 17% MSSA (*n* = 43) and 11.1% MRSA (*n* = 28)), Group D Streptococci (14.6%, *n* = 37), and *Proteus Mirabilis* (11.9%, *n* = 28).

A total of 35.4% (*n* = 606) underwent recurrent LEAs on the same limb, and 21.9% on the contralateral limb, 6.3% (*n* = 107) being major LEAs. Only 3.3% (*n* = 57) of participants had a history of revascularization procedures. The length of stay ranged from 1 to 71 days with an average of 6.26 days (CI95% 6.05–6.46), with significantly longer durations in the case of major LEAs (6.61 days (CI95% 6.31–6.9) vs. 6.11 days (5.85–6.38), *p* = 0.032). Upon admission, the patients presented hyperglycaemia, leucocytosis, and inflammatory syndrome (higher C-reactive protein) (Table 2).

The characteristics of the population are described in Table 1.

### 3.2. Major Amputations

**Figure 1:**
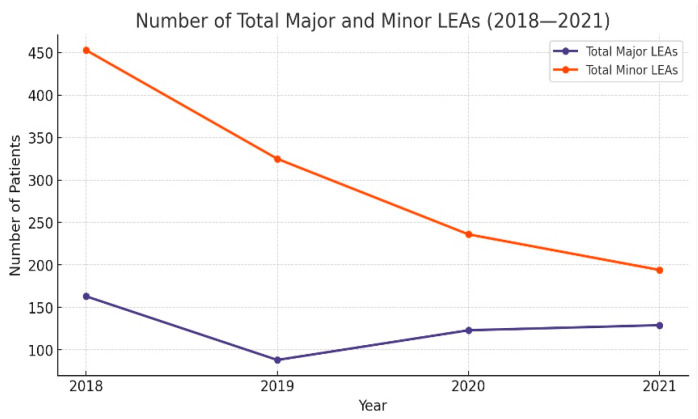
Absolute number of major and minor LEAs.

**Figure 2:**
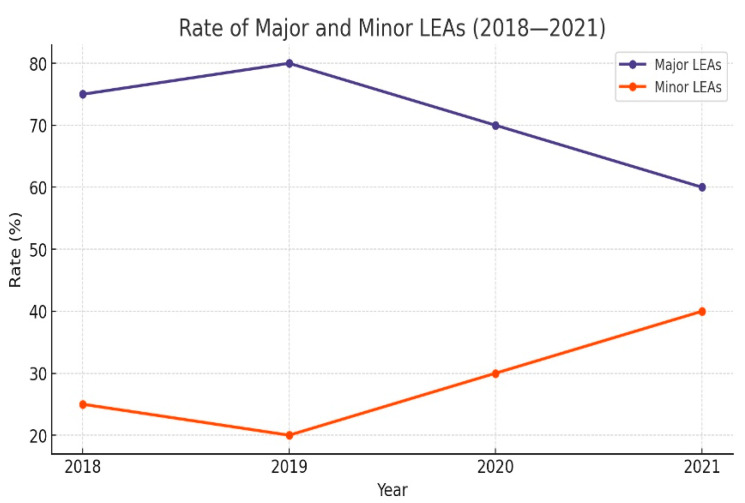
Rate of major and minor LEAs.

Approximately one-third of all LEAs were major (29.4%, *n* = 503). The total number of LEAs decreased during the period of observation; 616 LEAs were performed in 2018, and 323 LEAs in 2021 (*p* < 0.001) (Table 3). Even though numerically the number of major amputations decreased (Figure 1), the rate of major LEAs increased in 2020 and 2021 (Figure 2). The ratio of major amputations to minor was initially 0.41 and had an upward trend. We also noted a rising trend for the proportion of major amputations in both men (*p* < 0.001) and women (*p* = 0.004).

The most common was transtibial amputation at 18.9% (*n* = 323) (without gender differences). The number of transtibial amputations increased by 1.7 times between 2018 and 2021. This was followed by transfemoral amputations at 10.5% (*n* = 180); the proportion of transfemoral amputations increased by 1.18 times over the years. The majority of patients who underwent major LEAs were male, with an M/F ratio of 2.92, diagnosed with PAD (peripheral arterial disease) (75.1%, *n* = 378), PNP (polyneuropathy) (44.1%, *n* = 222), CKD (chronic kidney disease) (35%, *n* = 176), obesity (46.1%, *n* = 232), hypertension (76.5%, *n* = 385), and 39.1% current smokers (Table 4).

### 3.3. Minor Amputations

Between 2018 and 2021 were carried out 1208 minor LEAs (70.6%). For minor LEAs, the trend was descending for both genders (Table 3). The most common was ray amputation (including the toe and corresponding metatarsal bone) at 41.2% (*n* = 705). Minor LEAs decreased from 453 procedures in 2018 (73.5% of total LEAs) to 194 procedures in 2021 (60.1% of total LEAs). The patients who underwent minor LEAs were predominantly male (80%, *n* = 966), with an M/F ratio of 3.99, diagnosed with PNP (61.9%, *n* = 748), PAD (51%, *n* = 616), CKD (29.7%, *n* = 359), obesity (53.4%, *n* = 645), hypertension (74.4%, *n* = 899), with a high proportion of smoking (38.5%, *n* = 465) (Table 4).

### 3.4. Age

The mean age at admission increased from 64.034 ± 10.35 years in 2018 to 65.19 ± 9.43 years in 2021 (*p* = 0.03), being higher in those with major amputations (66.57 ± 9.39 years versus 63.68 ± 10.03 years, *p* < 0.001), both in women and men.

**Figure 3:**
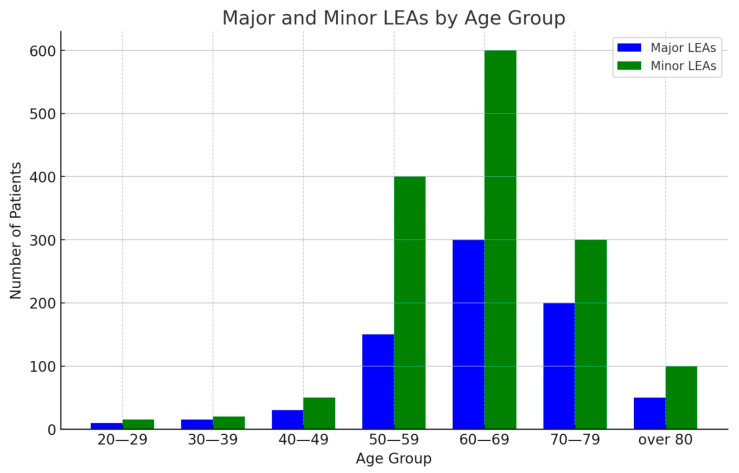
LEAs stratified by age group and type of amputation.

Overall, both minor and major amputations belonged to the age group 60–69 years (major LEAs *n* = 206, 41%; minor LEAs *n* = 530, 43.9%) (Figure 3); the second most prevalent age group was 70–79 years in women (31.1%, *n* = 115) and 50–59 years for men (24.2%, *n* = 324) (Figure 3).

### 3.5. Smoking

Current smoking status was noticed in 38.8% (*n* = 664) of the included subjects; the percentage of smokers increased year after year; significantly more men than women were smoking (44.2% versus 19.2%, *p* = 0.001). Similar proportion of smokers was found both in major (39.6% (*n* = 199)) and minor amputations (38.5%, *n* = 465). There were no differences remarked between smokers and nonsmokers regarding the type of intervention.

### 3.6. Type of Diabetes

There were twenty-nine patients with T1DM, and 34.5% (*n* = 10) underwent major amputations. In patients with T2DM, 29.3% (*n* = 493) suffered major amputations, and 70.7% (*n* = 1208) had minor amputations (*p* = 0.541). Amputation of the toe and the metatarsal bone was the most common minor LEA in T1DM (*n* = 11, 57.9%) and T2DM (*n* = 694, 58.35%). Transtibial amputation was the most frequent major LEA, both in type 1 and type 2 diabetes; in T1DM patients, there were six cases (60%), and in T2DM people, 316 (64.09%).

### 3.7. The Complications of Diabetes Mellitus

Peripheral arterial disease (PAD) (*n* = 994, 58.1%) and diabetic polyneuropathy (DPN) (*n* = 970, 56.7%) were the most prevalent diabetes-related complications in patients who underwent LEAs. Chronic kidney disease (CKD) was present in 31.3% (*n* = 535), and 9% had end-stage renal disease (ESRD) (*n* = 154). An increased proportion of patients had associated cardiovascular risk factors such as hypertension (*n* = 1284, 75%), obesity (*n* = 877, 51.3%), and dyslipidaemia (*n* = 516, 30.2%) (Table 5).

In univariate analysis, factors associated with major LEAs were age over 70, gender, sepsis, obesity, DPN, PAD, CKD, and leucocytosis. In multivariate analysis, patients with diabetes and sepsis, diabetic polyneuropathy, age over 65, and leucocytosis were more likely to undergo major amputation (Table 6). For minor amputation, in multivariate logistic regression, age over 65 years, sepsis, diabetic polyneuropathy, and leucocytosis could be considered independent risk factors (Table 6).

The total number of LEAs decreased significantly in the pandemic period (682 in pandemic era vs. 1029 in pre-pandemic period). This decrease was obtained mainly because of the reduction of minor LEAs (778 pre-pandemic vs. 430 in the pandemic era), while the number of major LEAs remained approximately constant (251 vs. 252). However, there was a statistically significant increase in the rate of major LEAs in the pandemic vs. pre-pandemic period (37% vs. 24.4%, *p* < 0.001). There were significantly higher percentages of transtibial and transfemoral amputations in the pandemic versus pre-pandemic period (24.9% vs. 14.8% and 12% vs. 9.6%, respectively). The patients who needed amputations in pandemic era had leucocytosis (55.6% to 46.5%, *p* < 0.001) and sepsis (28.9% to 17.5%, *p* < 0.001) more frequently than the patients who needed amputations in the pre-pandemic era (Table 7).

## 4. Discussion

In our four-year retrospective study, between January 2018 and December 2021, 1711 patients underwent an LEA, and 29.4% of all LEAs were major (*n* = 503). Our data are similar with the statistics published for Romania between 2015 and 2019, when major amputation represented 28.87% of the LEA-affected individuals [11].

Most of our patients were in their 6th decade, with a mean age of 64.53 ± 9.93 (range 26–93) years, similar to other studies [8,11].

Our results showed that men are disproportionately affected by LEAs, with a ratio of 3.62 (1341 vs. 370, *p* = 0.014), consistent with results from extensive studies available in the literature. Ezzatvar et al. published a meta-analysis that provided global estimates of diabetes-related amputation incidence from 2010 to 2020; the incidence of LEAs in males was approximately two-fold higher than in women [8]. In another cohort, men exhibited a higher prevalence of LEAs compared to women, irrespective of whether they had type 1 or type 2 diabetes mellitus [12].

There are several hypotheses for this difference. On the one side, the risk factors for LEA seem to be more prevalent in men: diabetic foot ulcers, peripheral artery disease, or tobacco consumption [8]. Our data likewise showed that more men were smoking (44.2% versus 19.2%, *p* = 0.001). On the other hand, in women of reproductive age, oestrogens could have a vascular and neural protective effect [13].

In our analysis, septic complications were most frequently the reason for LEA, wet gangrene and infected ulcers causing together 62.7% of the indications for LEA. Unlike other large studies that named infected ulcers as the leading cause for LEAs [8,14], in our analysis, wet gangrene (40.9%, *n* = 699) was the most frequent cause, with infected ulcers accounting for only 21.8% of the cases. One explanation may be that our patients were addressed later to the healthcare services, in more advanced stages of the disease.

The risk factors identified in our study are consistent with other results published in the literature [15,16,17]. In a meta-analysis including 6000 patients with diabetic foot infections, the predictors for LEAs were identified as male gender, smoking habits, a previous amputation, peripheral arterial disease, diabetic retinopathy, osteomyelitis, severe infections, gangrene/necrosis, and advanced scores in the International Working Group on the Diabetic Foot (IWGDF) (grades 3 or 4) and Wagner classifications (grades 4 or 5) [15]. Additionally, inflammatory markers such as leucocytosis, elevated CRP, and erythrocyte sedimentation rate levels were associated with an increased risk of amputation [15].

In another meta-analysis, which included 21 studies with 6505 participants, PAD and infection severity were among the strongest predictors of LEA [16]. Male sex, smoking, and a history of foot ulcers or amputations were also linked to increased risk [16]. Comorbidities, including osteomyelitis, neuropathy, lower body mass index, and delayed wound healing, were highlighted as contributing factors for LEAs [16]. Early detection and management of PAD and infection, as well as addressing modifiable factors like smoking, could significantly reduce the incidence of amputations and emphasize the importance of targeted interventions and risk stratification in preventing amputations among patients with DFUs [16].

In a more recent meta-analysis that included 9934 subjects, previously identified risk factors for lower extremity amputations (LEAs) were reconfirmed [17]. Male sex and older age were associated with a higher prevalence of LEAs [17]. Although, age and the type of diabetes mellitus were not identified as significant risk factors for lower extremity amputation in individuals with diabetic foot ulcers [17]. Smoking cessation and improved wound care, especially in cases of infection or gangrene, were emphasized as critical for reducing LEA risk [17].

The total number of LEAs decreased constantly throughout the analysed period; 616 LEAs were performed in 2018 and 323 LEAs in 2021 (*p* < 0.001). The total number of LEAs decreased mainly by a reduction in minor LEAs (778 pre-pandemic vs. 430 in the pandemic era), the number of major LEAs remaining approximately constant (251 vs. 252). However, we observed an increasing tendency in the rate of major LEAs among males and females compared to minor LEAs, which was statistically significant (*p* < 0.001). This could support the idea that the access of DFD-affected patients to healthcare services during the pandemic era could have been hampered, as from the patients needing amputation procedures, more individuals needed major LEAs, meaning that they ended up receiving treatment in a more advanced stage of disease. This idea could also be supported by the fact that the patients who needed amputations in the pandemic era had leucocytosis (55.6% to 46.5%, *p* < 0.001) and sepsis (28.9% to 17.5%, *p* < 0.001) more frequently than the amputees in the pre-pandemic era. The hypothesis of impaired access of DFD-affected patients to healthcare services caused by the COVID-19 pandemic is also supported by other authors [18,19].

Disparities seem to be caused not only by impaired access to healthcare services due to the COVID-19 pandemic but also by limited access to complex, expensive medical procedures in our country. For instance, in the Netherlands, the majority (70%) of people with major LEAs had a history of endovascular revascularisation before amputation [20]. On the contrary, only a small proportion of our cohort (3.3%, *n* = 57) had access to revascularization procedures before being subject to LEA. Revascularization, both endovascular and surgical, is a first-class recommendation for limb salvage in patients with chronic limb-threatening ischaemia [21].

A study analysing global variations in amputation rates, using data from 12 countries (primarily European), highlighted that countries with lower gross domestic product (GDP) and healthcare expenditures, such as Hungary and Slovakia, reported the highest rates of major amputations [22,23].

The strengths of this study are the inclusion of a large number of patients treated in a tertiary care centre from the largest city of Romania, a significant analysed period (4 years), including 2 pre-pandemic years and 2 pandemic years, and a direct comparison between the pre-pandemic and pandemic period.

The limitations of our study would be the lack of data regarding quality of life, the impact of different medications in the magnitude of necessary LEAs, possible other disparities significantly involved (such as socioeconomic or ethnic disparities), the severity of diabetes in LEA-affected individuals (possibly quantified by HbA1c, duration of diabetes, or intensity of default antidiabetic treatment), a possible correlation between specific risk factors and repeated need for LEA in the same patient, or the single-centre nature of the study.

This paper brings to attention new data regarding LEAs caused by diabetes mellitus, highlights the risk factors involved in the unfortunate evolution of diabetes-related foot disease, discusses the impact of the COVID-19 pandemic upon diabetes-affected individuals, and could be the basis of future studies aimed at reducing the burden of diabetes.

## 5. Conclusions

LEAs, in general, and in diabetes-affected individuals in particular, remain an important public health issue, with immense emotional implications for patients and a significant burden for health and social services. This study highlights the ongoing public health challenge posed by lower extremity amputations (LEAs) in patients with diabetes mellitus, exacerbated during the COVID-19 pandemic. In our study, a positive trend was observed, as the total number of LEAs constantly decreased throughout the analysed period. Despite a reduction in the total number of LEAs over the study period, major amputations increased significantly during the pandemic, likely reflecting delays in access to care and advanced disease stages at presentation. Identified key risk factors, including age over 65 years, leucocytosis, sepsis, and diabetic polyneuropathy, underscore the need for early detection and intervention in diabetic foot disease. Further studies, including more years of surveillance and more patients from more centres, could give us a better picture of the trends in amputation rates in diabetic foot disease.

## Figures and Tables

**Table 1:** General characteristics of the population that underwent an LEA.

Variables		2018	2019	2020	2021	Total	*p* *	*p* **
Age (years)	Total	64.034 ± 10.35	64.32 ± 10.17	64.88 ± 9.83	65.19 ± 9.43	64.5 ± 9.94	<0.001	ns
	Male	63.400 ± 9.63	63.81 ± 9.73	64.409 ± 9.7	64.510 ± 8.77	63.9 ± 9.53		ns
	Female	66.31 ± 11.14	66.301 ± 11.6	67.029 ± 9.92	67.750 ± 11.25	66.7 ± 11.02		ns
Gender	Male	465 (75.6%)	330 (79.9%)	291 (81.1%)	255 (78.9%)	1341 (78.4%)		ns
	Female	150 (24.4%)	83 (20.1%)	68 (18.9%)	68 (21.1%)	369 (21.6%)		ns
Environment	Rural	227 (37%)	144 (35%)	120 (33.4%)	118 (36.6%)	609 (35.7%)		ns
	Urban	387 (63%)	268 (65%)	239 (66.6%)	204 (63.4%)	1098 (64.3%)		ns
Type of diabetes	1	10 (1.6%)	5 (1.2%)	8 (2.2%)	6 (1.9%)	29 (1.7%)	ns	ns
	2	602 (98.4%)	407 (98.8%)	350 (97.8%)	316 (98.1%)	1675 (98.3%)	ns	ns
Smoking	Total	235 (38.1%)	140 (33.9%)	156 (43.5%)	133 (41.2%)	664 (38.8%)	0.04	0.04
	Male	210 (45.2%)	128 (38.8)	138 (27.4%)	117 (45.9%)	593 (44.2%)	ns	
	Female	25 (16.6%)	12 (14.5%)	18 (26.5%)	16 (23.5%)	71 (19.2%)	ns	
Duration of diabetes (years) #		14.93 ± 9.82 13 (12)	14.27 ± 9.17 13 (12)	13.30 ± 9.91 11.5 (15)	13.64 ± 8.98 12 (13)	14.29 ± 9.54 13 (12)	<0.001	ns
Length of stay (days) #		6 (4)	5 (4)	5 (3)	5 (3)	5 (3)	ns	0.001

The data have been presented as mean ± standard deviation (SD) for continuous normally distributed data or as median and interquartile range (marked with #). *p* *—between gender, *p* **—between years; ns, statistically nonsignificant.

**Table 2:** Laboratory parameters at admission.

	2018		2019		2020		2021		*p*
	Median	Inter-quartile range	Median	Inter-quartile range	Median	Inter-quartile range	Median	Inter-quartile range	
FPG (mg/dL)	189.5	138	174	117	187	166	200	151	ns
Serum creatinine (mg/dL)	0.96	0.64	0.92	0.61	0.95	0.63	1.02	0.66	ns
eGFR (mL/min/1.73 mp)	79	49.75	81	49	81	50	72.5	46.75	ns
Leucocyte (10 × 10^9^ /L)	12	7	12	7	13	8	14	8	<0.001
C-reactive Protein (mg/dL)	147.5	119.5	163.5	191.25	117	151	137.5	135.48	ns

Abbreviations: FPG, fasting plasma glucose; eGFR, estimated glomerular filtration rate. The data have been presented as median and inter-quartile range. ns, statistically nonsignificant.

**Table 3:** Number of LEAs during the study period, stratified by gender.

Gender	Type of LEAs	2018	2019	2020	2021	Total
Male	Major	115 (24.7%)	69 (20.9%)	96 (33%)	95 (37.3%)	375 (28%)
	Minor	350 (75.3%)	261 (79.1%)	195 (67%)	160 (62.7%)	966 (72%)
Female	Major	48 (31.8%)	19 (22.9%)	27 (39.7%)	34 (50%)	128 (34.6%)
	Minor	103 (68.2%)	64 (77.1%)	41 (60.3%)	34 (50%)	242 (65.4%)
Total	Major	163 (26.5%)	88 (21.3%)	123 (34.3%)	129 (39.9%)	503 (29.4%)
	Minor	453 (73.5%)	325 (78.7%)	236 (65.7%)	194 (60.1%)	1208 (70.6%)
All LEAs		616	413	359	323	1711

Abbreviations: LEA, lower extremity amputation. Variables are presented as percentages.

**Table 4:** Characteristics of patients with major and minor LEAs.

	Major LEAs (*n* = 503, 29.4%)	Minor LEAs (*n* = 1208, 70.6%)	*p*
Age (years)	66.57 ± 9.39	63.68 ± 10.03	ns
Gender, male	375 (74.6)	966 (80%)	0.014
Gender, female	128 (25.4)	242 (20%)	0.014
Type 1 diabetes	10 (2%)	19 (1.6%)	0.541
Type 2 diabetes	493 (98%)	1189 (98.4%)	ns
Smoking (*n*, %)	199 (39.6%)	465 (38.5%)	0.703
Hypertension (*n*, %)	385 (76.5%)	899 (74.4%)	0.391
Obesity (*n*, %)	232 (46.1%)	645 (53.4%)	0.001
Dyslipidaemia (*n*, %)	150 (29.8%)	366 (30.3%)	0.862
PAD (*n*, %)	378 (75.1%)	616 (51%)	<0.001
DPN	222 (44.1%)	748 (61.9%)	<0.001
PAD + DPN	160 (31.8%)	361 (29.9%)	0.454
CKD	176 (35%)	359 (29.7%)	0.034
ESRD	53 (10.5%)	51 (4.2%)	0.02
DR	46 (9.1%)	108 (8.9%)	0.926
Charcot foot	32 (6.4%)	26 (2.2%)	0.001
History of revascularisation	13 (2.6%)	44 (3.6%)	0.303
Main causes: Wet gangrene/Ischemia/Infected ulcers	233 (46.3%) 100 (19.9%) 51 (12%)	466 (38.6%) 104 (8.6%) 322 (26.7%)	
SGLT2 inhibitors	8 (1.6%)	37 (3.1%)	0.097

Abbreviations: LEA, lower extremity amputation; PAD, peripheral arterial disease; DPN, diabetic polyneuropathy; CKD, chronic kidney disease; ESRD, end-stage renal disease; DR, diabetic retinopathy. Variables are presented as percentages.

**Table 5:** Complications and comorbidities stratified by year and gender.

		2018		2019		2020		2021		Total	
Complications and Comorbidity		No. of Patients	(%)	No. of Patients	(%)	No. of Patients	(%)	No. of Patients	(%)	No. of Patients	(%)
DPN	Total	343	55.70%	225	54.50%	196	54.60%	206	63.80%	970	56.70%
	Men	270	58.10%	184	55.80%	161	55.30%	167	65.50%	782	58.30%
	Women	73	48.30%	41	49.40%	35	51.50%	39	57.40%	188	50.80%
PAD	Total	361	58.60%	214	51.80%	225	62.70%	194	60.10%	994	58.10%
	Men	266	57.20%	165	50.00%	184	63.20%	152	59.60%	767	57.20%
	Women	95	62.90%	49	59.00%	41	60.30%	42	61.80%	227	61.40%
DPN + PAD	Total	192	31.20%	96	23.20%	116	32.30%	117	36.20%	521	30.50%
	Men	143	30.80%	76	23.00%	98	33.70%	93	36.50%	410	30.60%
	Women	49	32.50%	20	24.10%	18	26.50%	24	35.30%	111	30.00%
CKD	Total	178	29.10%	127	30.80%	118	32.90%	111	34.40%	535	31.30%
	Men	133	28.60%	107	32.40%	93	32.00%	89	34.90%	422	31.50%
	Women	46	30.50%	20	24.10%	25	36.80%	22	32.40%	113	30.50%
ESRD	Total	59	9.60%	41	9.90%	31	8.60%	23	7.10%	154	9.00%
	Men	36	7.70%	29	8.80%	22	7.60%	15	5.90%	102	7.60%
	Women	23	15.20%	12	14.50%	9	13.20%	8	11.80%	52	14.10%
Hypertension	Total	455	73.90%	292	70.70%	280	78.00%	257	79.60%	1284	75.00%
	Men	331	71.20%	227	68.80%	218	74.90%	197	77.30%	973	72.60%
	Women	124	82.10%	65	78.30%	62	91.20%	60	88.20%	311	84.10%
Dyslipidaemia	Total	137	22.20%	111	26.90%	119	33.10%	149	46.10%	516	30.20%
	Men	106	22.80%	92	27.90%	94	32.30%	118	46.30%	410	30.60%
	Women	31	20.50%	19	22.90%	25	36.80%	31	45.60%	106	28.60%
BMI over 30 kg/m^2^	Total	294	47.70%	193	46.70%	200	55.70%	190	58.80%	877	51.30%
	Men	229	49.20%	150	45.50%	156	53.60%	152	59.60%	687	51.20%
	Women	65	43.00%	43	51.80%	44	64.70%	38	55.90%	190	51.40%

Abbreviations: LEA, lower extremity amputation; DPN, diabetic polyneuropathy; PAD, peripheral arterial disease; CKD, chronic kidney disease; ESRD, end-stage renal disease; BMI, body mass index. Variables are presented as percentages.

**Table 6:** Factors associated with LEAs.

	B	S.E.	*p*	OR	95% C.I.	
					Lower	Upper
Major LEAs						
Sepsis	0.851	0.142	<0.001	2.342	1.772	3.094
DPN	0.597	0.117	<0.001	1.817	1.445	2.285
Leucocytosis	0.081	0.011	<0.001	1.084	1.061	1.107
Age over 65 years	0.032	0.006	<0.001	1.032	1.020	1.045
Minor LEAs						
Sepsis	−1.021	0.139	<0.001	0.360	0.274	0.473
PAD	1.015	0.127	<0.001	2.760	2.154	3.537
DPN	−0.560	0.116	<0.001	0.571	0.455	0.717
Leucocytosis	0.708	0.126	<0.001	2.029	1.586	2.596
Age over 65 years	0.348	0.137	0.011	1.416	1.084	1.851

Abbreviations: LEA, lower extremity amputation; DPN, diabetic polyneuropathy; PAD, peripheral arterial disease. B, standard beta coefficient; SE, standard error; OR, odds ratio; CI, confidence interval.

**Table 7:** Number and type of LEAs in pre-pandemic vs. pandemic period.

	Pre-Pandemic	Pandemic	*p*
T2DM	1014 (98.5%)	668 (97.9%)	0.347
Minor LEAs	778 (75.6%)	430 (63%)	<0.001
Major LEAs	251 (24.4%)	252 (37%)	<0.001
Total LEAs	1029 (60.1%)	682 (39.9%)	<0.001
Sepsis	180 (17.5%)	197 (28.9%)	<0.001
Leukocytosis	478 (46.5%)	379 (55.6%)	<0.001
Toe amp	225 (21.9%)	124 (18.2%)	ns
TMT and toe	466 (45.3%)	239 (35%)	<0.001
TMT amp	87 (8.5%)	67 (9.8%)	ns
Transtibial amp	152 (14.8%)	170 (24.9%)	<0.001
Transfemoral amp	99 (9.6%)	82 (12%)	<0.001

Abbreviations: T2DM, type 2 diabetes mellitus; LEA, lower extremity amputation; TMT, transmetatarsal; amp, amputation. ns, statistically nonsignificant.

## Data Availability

The original contributions presented in the study are included in the article; further inquiries can be directed to the corresponding authors.
